# Supplementary material for: Dynamical barrier and isotope effects in the simplest substitution reaction via Walden inversion mechanism
Source: Nat Commun. 2017 Feb 22;8:14506. doi: 10.1038/ncomms14506 (PMC5336572; doi:10.1038/ncomms14506)
Supplement: Supplementary Information — Supplementary Figures, Supplementary Methods and Supplementary References. [file ncomms14506-s1.pdf]

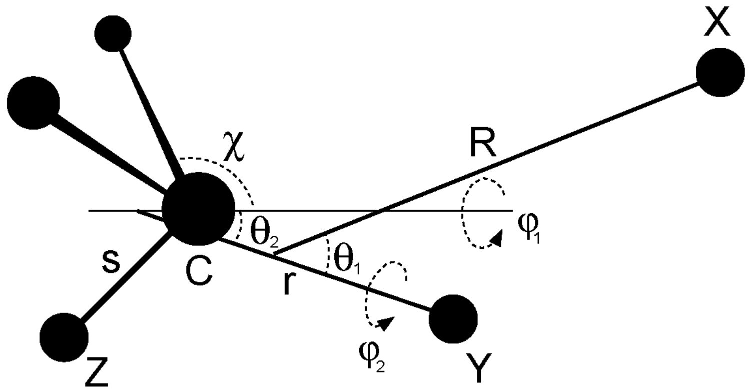


Supplementary Figure 1. The eight-dimensional Jacobi coordinates for the $X+YCZ_3$.

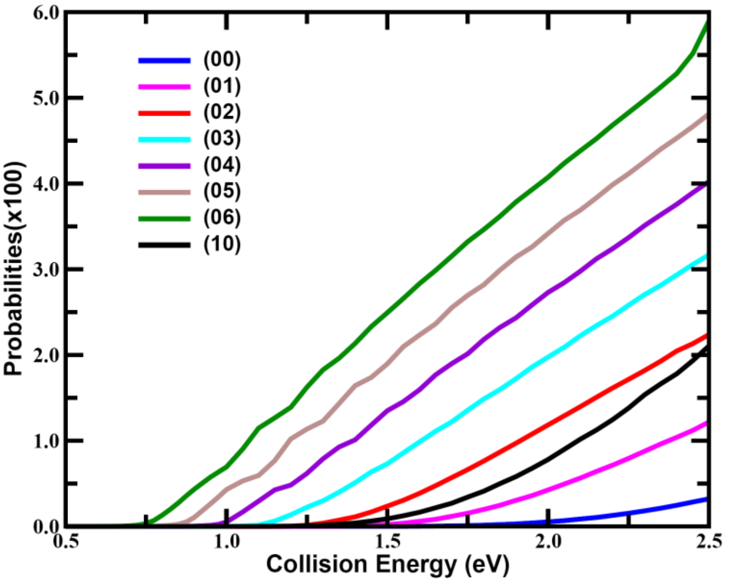


Supplementary Figure 2. Same as Fig. 3(a) except for more initial states.

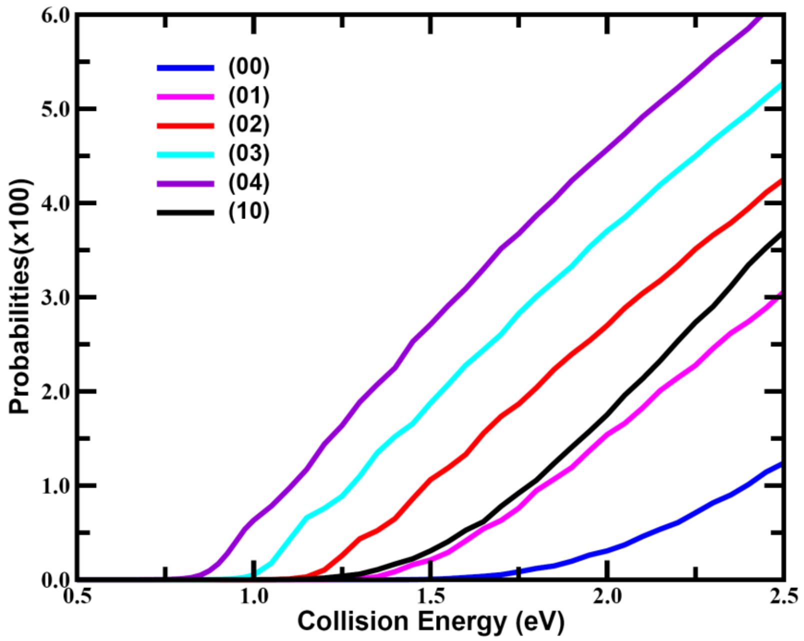


Supplementary Figure 3. Total reaction probabilities for a number of initial vibrational states for reaction (1) as a function of collision energy

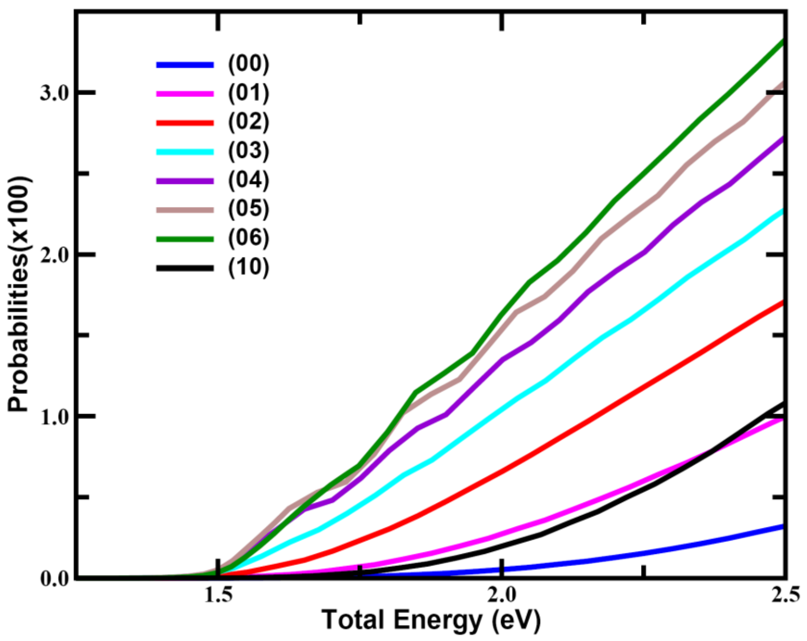


Supplementary Figure 4. Same as Fig. 3(b) except for more initial states.

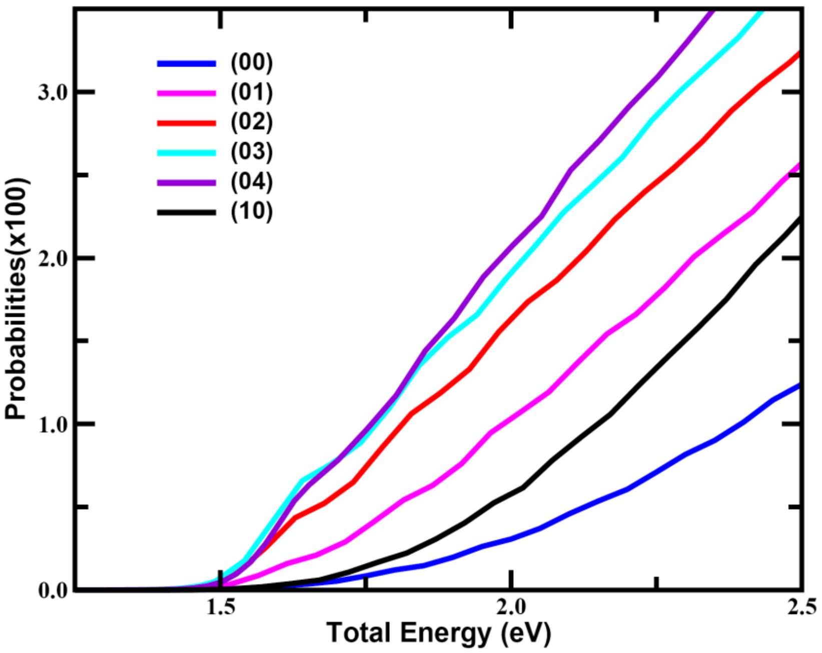


Supplementary Figure 5. Total reaction probabilities for a number of initial vibrational states for Reaction (1) as a function of total energy measured from the ground state energy of $CH_4$.

**Supplementary Methods**

**A. Reduced dimensional Hamiltonian and basis set expansion**

The time-dependent quantum wave packet calculations employed the eight dimensional (8D) model for the X+$YCZ_3$ reaction by restricting the non-reacting $CZ_3$ group under $C_{3V}$ symmetry[1,2]. The 8D model Hamiltonian in the Jacobi coordinates $(R, r, s, \chi, \theta_1, \theta_2, \varphi_1, \varphi_2)$ shown in Supplementary Figure 1 can be written as[3]

$$\hat{H} = -\frac{1}{2\mu_R}\frac{\partial^2}{\partial R^2} - \frac{1}{2\mu_r}\frac{\partial^2}{\partial r^2} + \frac{\left(\hat{J}_{tot} - \hat{J}\right)^2}{2\mu_R R^2} + \frac{\hat{l}^2}{2\mu_r r^2} + \hat{K}_{CZ_3}^{vib} + \hat{K}_{CZ_3}^{rot} + V(R, r, s, \chi, \theta_1, \theta_2, \varphi_1, \varphi_2),$$

where $\mu_R$ is the reduced mass of X and $YCZ_3$, $\mu_r$ is the reduced mass of Y and $CZ_3$; $R$ is the distance from the center of mass of $YCZ_3$ to X; $r$ is the distance from the center of mass of $CZ_3$ to Y; $s$ is the bond length of CZ; $\chi$ is the angle between a CZ bond and the $C_{3V}$ symmetry axis of $CZ_3$. $\hat{J}_{tot}$ is the total angular momentum operator of the system; $\hat{J}$ is the rotational angular momentum of $YCZ_3$; and $\hat{l}$ is the orbital angular momentum operator of Y with respect to $CZ_3$. $\hat{K}_{CZ_3}^{vib}$ and $\hat{K}_{CZ_3}^{rot}$ are the vibrational and rotational kinetic energy operators of $CZ_3$, respectively, and are defined as

$$\hat{K}_{CZ_3}^{vib} = -\frac{\hbar^2}{2s^2}\left(\frac{cos^2\chi}{\mu_\chi} + \frac{sin^2\chi}{\mu_s}\right)\frac{\partial^2}{\partial \chi^2} - \frac{\hbar^2}{s^2}\left(\frac{1}{\mu_s} - \frac{1}{\mu_\chi}\right) sin\chi cos\chi \frac{\partial}{\partial \chi}$$

and

$$\hat{K}_{CZ_3}^{rot} = \frac{1}{2I_A}\hat{j}^2 + \left(\frac{1}{2I_C} - \frac{1}{2I_A}\right)\hat{j}_z^2,$$

where $\hat{j}^2$ and $\hat{j}_z^2$ are, respectively, the angular momentum operator for the $CZ_3$ group and its projection of the C3 axis, $\mu_\chi$ and $\mu_s$ are related to the mass of atoms C and Z, with $\mu_\chi = 3m_Z$ and $\mu_s = 3m_C m_Z/(m_C + 3m_Z)$, $I_A$ and $I_C$ are the rotational inertia of $CZ_3$, defined as

$$I_A = \frac{3}{2}m_Z s^2\left(sin^2\chi + \frac{2m_C}{m_C + 3m_Z}cos^2\chi\right)$$

and

$$I_C = 3m_Z s^2 sin^2\chi.$$

With the length of the C-Z bond fixed, the seven dimensional time-dependent wave function is expanded in terms of basis functions of $R, r, \chi$ and the body-fixed (BF) total angular momentum eigenfunctions as

$$\Psi^{J_{tot}M\varepsilon} = \sum_{n,v_r,v_\chi} \sum_{K\,Jljk} C^{J_{tot}MK\varepsilon}_{nv_rv_\chi Jljk}(t)\, F^{v_r}_n(R)\phi_{v_r}(r)\phi_{v_\chi}(\chi)\phi^{J_{tot}MK\varepsilon}_{Jljk}(\hat{R},\hat{r},\hat{s})$$

where $C^{J_{tot}MK\varepsilon}_{nv_rv_\chi Jljk}(t)$ are time-dependent coefficients, $n$, $v_r$, and $v_\chi$ are the labels for the basis functions in $R$, $r$, and $\chi$ coordinates, respectively. $F^{v_r}_n(R)$ are sine basis functions for $R$ which are dependent on $v_r$ for their spatial ranges to separate interaction region from asymptotic region. The basis functions $\phi_{v_r}(r)$ and $\phi_{v_\chi}(\chi)$ are obtained by solving the following one-dimensional reference Hamiltonians for $r$, $\chi$ respectively,

$$h_r(r) = -\frac{1}{2\mu_r}\frac{\partial^2}{\partial r^2} + V^{ref}_r(r)$$

and

$$h_\chi(\chi) = K^{vib}_{CZ} + V^{ref}_\chi(\chi)$$

where $V^{ref}_r(r)$ and $V^{ref}_\chi(\chi)$ are the corresponding reference potentials.

The BF total angular momentum basis functions $\phi^{J_{tot}MK\varepsilon}_{Jljk}$ are defined as

$$\phi^{J_{tot}MK\varepsilon}_{Jljk}(\hat{R},\hat{r},\hat{s}) = \sqrt{\frac{1}{2(1+\delta_{\bar{K}0}\delta_{k0})}}[\bar{D}^{J_{tot}}_{MK}(\hat{R})Y^{JK}_{jlk}(\hat{r},\hat{s})$$
$$+\varepsilon(-1)^{J_{tot}+J+l+j+k}\bar{D}^{J_{tot}}_{M-K}(\hat{R})Y^{J-K}_{jl-k}(\hat{r},\hat{s})]$$

where $\varepsilon$ is the parity of the system and $\bar{D}^{J_{tot}}_{MK}(\hat{R})$ is defined as

$$\bar{D}^{J_{tot}}_{MK}(\hat{R}) = \sqrt{\frac{2J_{tot}+1}{8\pi^2}}D^{*J_{tot}}_{MK}(\alpha,\beta,\gamma)$$

with *M* and *K* being the projection of total angular momentum $\hat{J}_{tot}$ on the z-axis of the space-fixed and body-fixed frames, respectively. $\bar{D}^{J_{tot}}_{MK}(\hat{R})$, the Wigner rotation matrix, depends on Euler angles which rotate the space-fixed frame onto the body-fixed frame and are the eigenfunctions of $\hat{J}^2_{tot}$. The spherical harmonics

$Y_{jlk}^{JK}(\hat{r},\hat{s})$ are given by

$$Y_{jlk}^{JK}(\hat{r},\hat{s}) = \sum_m \bar{D}_{Km}^J(\hat{r}) \sqrt{\frac{2l+1}{2J+1}} < jml0|Jm > \bar{D}_{mk}^j(\hat{s})$$

where $\bar{D}_{Km}^J(\hat{r})$ depends on Euler angles which rotate the $XYCZ_3$ body-fixed frame onto the $YCZ_3$-fixed frame, and $\bar{D}_{mk}^j(\hat{s})$ depends on Euler angles which rotate the $YCZ_3$-fixed frame onto the $CZ_3$-fixed frame,

$$\bar{D}_{Km}^J(\hat{r}) = \sqrt{\frac{2J+1}{4\pi}} D_{Km}^{*J}(0,\theta_1,\varphi_1),$$

$$\bar{D}_{mk}^j(\hat{s}) = \sqrt{\frac{2j+1}{4\pi}} D_{mk}^{*j}(0,\theta_2,\varphi_2).$$

**B. Cumulative reaction probability and thermal rate constant**

The cumulative reaction probability $N_i(E)$ for a specific initial wave packet for a whole energy range can be calculated from the time-independent wave function on a second surface located at $r = r_s$,

$$N_i(E) = \frac{\hbar}{\mu_r} Im(\langle\psi_{iE}|\psi'_{iE}\rangle)|_{r=r_s},$$

where $\psi_{iE}$ and $\psi'_{iE}$ are the time-independent wave function and its first derivative in $r$. The time-independent wave function $\psi_{iE}$ is constructed by a Fourier transformation of the initial time-dependent wave packet as

$$|\psi_{iE}\rangle = \sqrt{\lambda}\int_{-\infty}^{+\infty} e^{i(E-H)t\hbar} |\Psi_i(0)\rangle dt,$$

where $\lambda$ is the eigenvalue of the flux operator with eigenfunction $|+\rangle$. From $N_i(E)$, the total cumulative reaction probability $N(E)$ can be simply calculated as

$$N(E) = \sum_i N_i(E).$$

In this study, we only calculate the cumulative reaction probability $N(E)$ for $J_{tot} = 0$, $N_{J_{tot}=0}(E)$. Under the $J$ shifting approximation[4,5] the thermal rate constant can be obtained from $N_{J_{tot}=0}(E)$ as

$$k(T) = \frac{Q_{rot}^{\ddagger}}{2\pi Q_r(T)} \int_0^{\infty} dE e^{-E/kT} N_{J_{tot}=0}(E),$$

where $Q^{\ddagger}_{rot}$ is the rotational partition function for the $CH_5$ complex at the transition state point and $Q_r(T)$ is the partition function of the reactants as

$$Q_r(T) = Q_{rot,CH_4}(T) Q_{vib,CH_4}(T) Q_{trans,H+CH_4}(T),$$

$Q_{trans,H+CH_4}(T)$ is the partition function of the relative translational motion of the two reactants, $Q_{vib,CH_4}(T)$ is the vibrational partition function of methane, and $Q_{rot,CH_4}(T)$ is the rotational partition function,

$$Q_{rot}(T) = \frac{\sqrt{\pi}}{\sigma}\sqrt{\frac{T^3}{\Theta_a \Theta_b \Theta_c}},$$

$$\Theta_i = \frac{\hbar^2}{2 I_i k_B},$$

$\sigma$ is the symmetry number.

**C. Numerical parameters**

The wavefunction is propagated using the split-operator propagator. An L-shaped wavefunction expansion for $R$ and r was used to reduce the size of the basis set[3]. For the H+$CH_4$ system, a total number of 120 sine basis functions covering a range from 0.5 to 15.0 bohrs were used for $R$ with 80 grid points in the interaction region. For the $r$ dimension, 35 basis functions were used in the range of [1.0,5.0] bohrs in the interaction region, while 6 basis functions were used in the asymptotic region. The length of the non-reacting CH bond was fixed at 2.06 bohr, which is the equilibrium CH distance of the reactant molecule. The number of basis functions for the umbrella motion was 17 for both the ground and excited initial states. The rotational basis functions was constrained by the parameters, $J_{max}$=90, $l_{max}$=60, $j_{max}$=30, and $k$=6. Under the CS approximation we only need to include the $K$=0 rotational basis functions in our calculation. With the parity and $C_{3V}$ symmetry of the $CH_3$ group taking into account, the size of rotational basis functions was ~120,000 for the A symmetry. The center of the initial Gaussian wave packet was located at $R_0$=13.0 bohrs with the width $\delta$=0.2 bohr, and the central energy was $E_0$=1.2 eV. We propagated the wave packets for 7000 a.u. of time with a time step of 10 to get fully converged reaction probabilities measured at $r$=3.2 bohrs. We calculated the total reaction probabilities for $J_{tot}$ up to 40 to converge the integral cross section at the

collision energy of 2.5 eV.

In the transition state wave packet calculations[6], the vibrational eigenfunctions on the first dividing surface (through the saddle point) were solved using a smaller basis sets than those described above. A total number of 20 sine basis functions covering a range from 0.5 to 3.5 bohrs were used for $R$. A total number of 40 transition-state wave packets were used in order to obtain the cumulative reaction probabilities shown in Fig. 3(c).

**Supplementary References**


[1] Palma, J. & Clary, D. C. A quantum model Hamiltonian to treat reactions of the type $X+YCZ_3 \rightarrow XY+CZ_3$: Application to $O(^3P)+CH_4 \rightarrow OH+CH_3$. *J. Chem. Phys.* **112**, 1859-1867 (2000).

[2] Yang, M., Zhang, D. H. & Lee, S. -Y. A seven-dimensional quantum study of the $H+CH_4$ reaction. *J. Chem. Phy.* **117**, 9539-9542 (2002).

[3] Yang, M., Lee, S. –Y. & Zhang, D. H. Seven-dimensional quantum dynamics study of the $O(^3P)+CH_4$ reaction. *J. Chem. Phys.* **126**, 064303 (2007).

[4] Bowman, J. M. Reduced dimensionality theory of quantum reactive scattering *J. Phys. Chem.* **95**, 4960-4968 (1991).

[5] Wu, T., Werner, H. –J. & Manthe, U. First-principles theory for the $H+CH_4 \rightarrow H_2+CH_3$ Reaction. *Science* **306**, 2227-2229 (2004).

[6] Zhang, L., Lu, Y., Lee, S. –Y. & Zhang, D. H. A transition state wave packet study of the $H+CH_4$ reaction. *J. Chem. Phys.* **127**, 234313 (2007).
